# A Generalized Model to Estimate the Statistical Power in Mitochondrial Disease Studies Involving 2×*k* Tables

**DOI:** 10.1371/journal.pone.0073567

**Published:** 2013-09-27

**Authors:** Jacobo Pardo-Seco, Jorge Amigo, Wenceslao González-Manteiga, Antonio Salas

**Affiliations:** 1 Unidade de Xenética, Departamento de Anatomía Patolóxica e Ciencias Forenses, and Instituto de Ciencias Forenses, Grupo de Medicina Xenómica (GMX), Facultade de Medicina, Universidade de Santiago de Compostela, Galicia, Spain; 2 Departamento de Estadística e Investigación Operativa, Universidade de Santiago de Compostela, Santiago de Compostela A Coruña, Spain; Instituto de Investigación Hospital 12 de Octubre, Spain

## Abstract

**Background:**

Mitochondrial DNA (mtDNA) variation (i.e. haplogroups) has been analyzed in regards to a number of multifactorial diseases. The statistical power of a case-control study determines the *a priori* probability to reject the null hypothesis of homogeneity between cases and controls.

**Methods/Principal Findings:**

We critically review previous approaches to the estimation of the statistical power based on the restricted scenario where the number of cases equals the number of controls, and propose a methodology that broadens procedures to more general situations. We developed statistical procedures that consider different disease scenarios, variable sample sizes in cases and controls, and variable number of haplogroups and effect sizes. The results indicate that the statistical power of a particular study can improve substantially by increasing the number of controls with respect to cases. In the opposite direction, the power decreases substantially when testing a growing number of haplogroups. We developed mitPower (http://bioinformatics.cesga.es/mitpower/), a web-based interface that implements the new statistical procedures and allows for the computation of the *a priori* statistical power in variable scenarios of case-control study designs, or e.g. the number of controls needed to reach fixed effect sizes.

**Conclusions/Significance:**

The present study provides with statistical procedures for the computation of statistical power in common as well as complex case-control study designs involving 2×*k* tables, with special application (but not exclusive) to mtDNA studies. In order to reach a wide range of researchers, we also provide a friendly web-based tool – mitPower – that can be used in both retrospective and prospective case-control disease studies.

## Introduction

The mitochondrion produces most of the ATP in the cell, an energy source on which almost all physicochemical processes depend. Each cell contains dozens or hundreds of mtDNA genomes that are inherited as a single haplotypic block from the mother to the offspring. Germ-line mutations accumulate on top of existing haplotypes, and these haplotypes aggregate in human populations according to their demographic histories. Due to the particularities of the mtDNA molecule (i.e. matrilineal inheritance and lack of recombination [1]), it is straightforward to reconstruct phylogenetic relationships between human haplotypes [2]–[4]. Phylogenetically related haplotypes in the population are commonly grouped into clusters or haplogroups [5]. Thus, haplogroups represent branches of the mtDNA phylogeny, and the set of diagnostic variants defining these clades are popularly known as the sequence motif [6], [7]. Screening for these variants in a given mtDNA molecule can provide sufficient information to allocate a particular mtDNA genome into a given haplogroup [8]–[10].

In the last few years, a huge number of studies have been conducted addressing the presumable association of mtDNA haplogroups with different complex diseases, including cancer [11], [12], Alzheimer [13], [14], Parkinson [15]–[17], schizophrenia [18]–[21], infectious diseases [22], [23], diabetes [24], LHON [25], etc. Most of these disease studies are population-based, that means, the mtDNA variability is compared between cohorts of cases and representative healthy control (case-control studies), where the statistically significant over-representation of a given variant in cases regarding controls might point to a biological association of this variant with the disease.

Statistical procedures are important in order to understand the presumable relationship between mtDNA haplogroups and diseases. Estimating *a priori* statistical power is fundamental in case-control association studies given that this is the way to evaluate to what extent a positive finding is likely to be not at random. However, case-control association studies targeting the mtDNA variation [22] rarely compute power mainly due to the lack of the statistical procedures that are necessarily different to those employed using autosomal DNA markers. To the best of our knowledge, only Samuels et al. [26] investigated the issue of statistical power in regards to cases-control mtDNA studies involving 2×*k*. These authors used a simulation-based permutation test (Monte-Carlo) in order to estimate power calculations for prospective case-control studies. According to these authors, very large cohorts are needed to reliably detect and association between mtDNA haplogroups and complex diseases. This study however only deals with the restricted scenario where the number of cases equals the number of controls. The particular biological application of Sánchez et al. [27] on a mtDNA case has to do specifically with 2×3 tables, comparing a RFLP polymorphism (binary) and the three genotypes derived from a biallelic albumin marker.

Several software packages and statistical procedures were designed for the calculation of statistical power and sample size. Most of the procedures developed to date can only deal with 2×2 tables (the great majority) or 2×3 tables [27] (**Table S1**). Thus, most of the software packages have been designed for the estimation of power or/and sample size in the most common scenario involving allele frequencies deriving from autosomal binary markers (SNPs), that is, involving allele or genotype association tests. Only two software packages, namely G*Power 3 [28] and Pass 12 [29], are able to treat tables *r*×*k*; however, these two packages only deal with scenarios where the number of cases equals number of controls. Finally, osDesign [30] is based on logistic regression, and although it can deal with *r*×*k* tables it does not allow estimating samples sizes.

In the present study we consider more general case-control disease scenarios involving any number of cases and controls and 2×*k* tables. For instance, it is a common situation that only a limited number of patients can be recruited in a particular study; however, an increase in the number of controls could contribute to reach a reasonable statistical power. The model elaborated in the present study is based on simulations (Monte Carlo method) as a way to estimate the statistical power in case-control studies where there is interest in investigating the presumable relationship between a certain disease and a number of mtDNA haplogroups (or haplotypes or mtDNA SNPs [mtSNPs]). We consider the frequency of the risky allele or haplogroup in controls (*p_0_*) and in cases (*p_1_*), and the difference between these two parameters is proportionally distributed to the frequencies of the remaining allele or haplogroup categories in cases. In addition, a web-tool named mitPower has been also developed to implement all the statistical procedures developed in the present study.

## Methods

### Data simulation

We first build 2×*N_H_* (in general, 2×*k*) tables (10,000 simulations), where 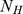 denotes the number of haplogroups considered (but could also be any number of haplotypes or mtSNPs). Two multinomial samples are used to build the contingency tables, taking as frequencies the estimated frequencies, and as size, the number of controls and cases of our study. In the simulated tables the row variable represents the status of case or control, while the columns represent the allele variables or haplogroups into which individuals are sorted. For the sake of simplicity, *N_H_* was set up to 11 (unless otherwise stated) but the method and mitPower has been designed to accept any number of haplogroups. As done in Samuels et al. [26], the following 11 haplogroup frequencies were considered as example: H (41%), I (2%), J (11%), K (8%), M (1%), T (13%), U (15%), V (13%), W (2%), X (2%) and a residual haplogroup (2%).

The power values obtained using MitPower have been validated with other tools (Table S1) in comparable scenarios that consider 2×2 tables and equal numbers of cases and controls. MitPower was additionally validated for 2×3 tables with the procedure shown by Sánchez et al. [27].

### Statistical analysis

**Figure 1:**
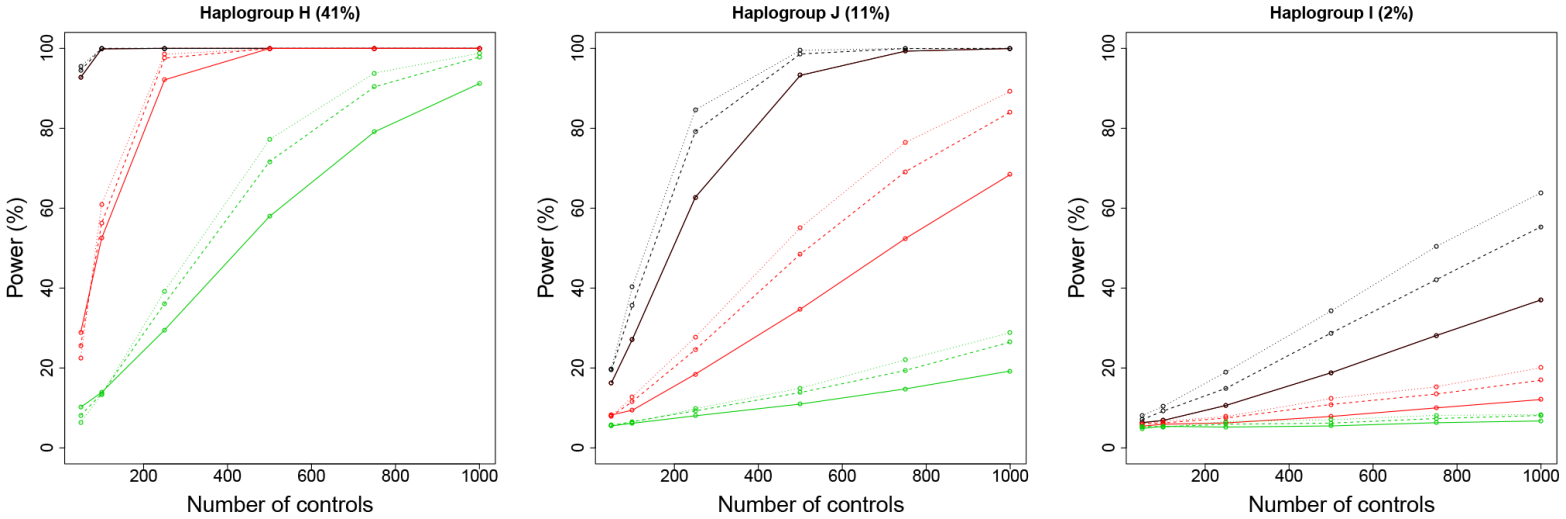
Representation of power values for three haplogroups (H, J. and I) as a function of the number of cases and using the Chi-square test (significance level α = 0.05). Colors indicate different deviations from the null hypothesis; thus, black represents a frequency in cases 100% higher than in controls, red represents an increment of 50%, and green an increment of 25% (with the difference distributed proportionally between the remaining non-risky haplogroups). The different lines indicate different case-control odds. The continuous line denotes an odd control-case of 1∶1, the dotted line of 2∶1, and the pointed line of 3∶1. Frequencies in controls for each haplogroup are indicated above each plot. Note that the results can be directly comparable with Samuels et al. [26] (see their **Figure** 1) when number of cases equals number of controls.

First, the computation of the probabilities of 2×2 contingency tables is the best option to test the homogeneity of control and case sample populations; however, the computational requirements increase with the dimension of the contingency tables. A way to overcome this problem is to implement a Fisher's exact test that estimates the probability of a contingency table using a Monte Carlo simulation approach. On the other hand, the Chi-squared statistic is computationally feasible for 2×*k* tables being *k* any entire positive number. Both tests yield very similar results (**Figure 1** and **Figure S1**) and both are implemented in MitPower. From here onwards, we have used the Chi-square statistic, which compares the values obtained in our contingency tables against the values expected under the null hypothesis of homogeneity.

First, we generate a number of tables 2×*N_H_* under a given hypothesis. A random number between 0 and 1 is generated using the R function *runif*, and this number is used as the seed for simulations. Power value estimators are obtained as the percentage of simulated tables with *P*-value below a fixed significance level.

In order to obtain the *P*-value for each simulated table, the distribution of our statistic has to be known. This distribution is approached here using two alternative procedures: the asymptotic and the permutation approach. The former approach is based on the asymptotic distribution of the Chi-square statistics. Note that some authors argued [31] that an increase of the false-positive rate occurs when the Cochran's rule is not verified; so the asymptotic approximation should be considered acceptable when the Cochran's rule is verified [31]; that means that a contingency table cannot contain expected values below one, and that no more than 20% of the expected value can be below five. The permutation method aims to overcome this problem. First, a large number of permuted tables of our initial data (contingency tables) are generated, with the only restriction that total sums by rows and columns have to remain constant. For each of these permutations, the Chi-square statistic is computed, and the *P*-value is obtained as the proportion of permutations with a Chi-square statistics higher than the statistics in the original data set [32], [33]. There are several ways to obtained permuted tables [32], [34], [35], and we chosen the method provided by the function *chisq.test* [36].

Theoretically, the asymptotic and the permutation approaches should have similar values as the sample size increases [37]. Some experiments have been done in this direction (**Table 1**, and see text below) in order to corroborate this expectation. Along the simulation experiments carried out in the present manuscript, the permutation method was preferred given that it generally performs better than the asymptotic one (see below).

**Table 1:** Estimates of statistical power (%) under the null hypothesis using the asymptotic distribution *versus* the permutation procedure, and elapsed computational times (in seconds) under different simulation scenarios.

Numberof ST	Number of cases	Asymptotic distribution	ElapsedTime	Permutations(1,000)	ElapsedTime	Permutations(10,000)	Elapsed Time
1,000	100	3.54	0.11	5.37	1.95	5.25	17.18
	200	4.29	0.09	5.21	2.50	5.42	22.07
	300	4.91	0.11	5.72	2.76	5.72	24.02
	400	3.80	0.10	4.00	2.90	4.00	25.59
	500	3.60	0.11	4.10	3.03	3.80	26.85
	600	4.50	0.10	5.20	3.19	4.80	28.10
	700	5.60	0.11	5.80	3.28	5.90	29.24
	800	5.20	0.09	5.50	3.41	5.40	30.31
	900	5.10	0.11	5.30	3.48	5.10	31.13
	1000	4.10	0.10	4.20	3.77	4.10	32.21
10,000	100	3.25	0.93	4.92	20.28	4.78	175.74
	200	4.00	1.13	4.78	33.21	4.81	280.80
	300	4.72	1.00	5.20	28.14	5.10	273.57
	400	4.72	1.32	4.68	30.00	4.74	290.17
	500	4.75	1.26	5.08	49.77	5.06	314.36
	600	4.73	1.03	4.98	32.15	4.93	312.73
	700	5.04	1.33	5.26	33.69	5.30	334.70
	800	5.27	1.23	5.57	50.14	5.41	330.14
	900	4.95	1.34	5.04	35.72	5.18	349.69
	1000	5.07	1.22	5.35	53.64	5.19	348.52

Estimates were computed for 1,000 and 10,000 simulated tables (ST), number of cases equal to number of controls, and level of significance α = 0.05 (therefore, estimated power values should be close to 5%). Time estimates were obtained using an Intel® Core™ I5 3.1 GHz. These values were averaged over ten simulations each.

All the computations were carried out in R (http://www.r-project.org/), and using the functions *chisq.test*, *fisher.test*, and *pchisq* of package *stats*.

### mitPower: a web interface to estimate statistical power in 2×k tables

mitPower is a web-based tool (http://bioinformatics.cesga.es/mitpower/) that allows estimating the statistical power in case-control association disease studies. Several other utilities are available in mitPower such as the estimation of: (i) the *a posteriori* statistical power, (ii) the sample size needed in order to reach a given statistical power, and (iii) the minimum deviation from the null hypothesis (of no association) detectable under a given statistical power (expressed as *OR* and haplogroup frequency in cases).

The software mitPower allows using two calibration methods: the asymptotic and the permutation procedure. The permutation procedure can be computationally demanding (see below) so the asymptotic procedure might be more convenient for complex scenarios.

The mitPower web interface is written in PHP, allowing users to enter their inputs through an HTML form. All the mitPower analyses are executed using R scripts (see above), which are called from the interface through *Rscript* and run at the web server. Their output is ultimately formatted for web display again by the PHP interface. Results links are kept for 24 hours in the server.

The underlying R scripts in mitPower run on a web server hosted by the Supercomputing Center of Galicia (CESGA; http://www.cesga.es) located in Santiago de Compostela (Galicia, Spain).

## Results and Discussion

Two procedures were followed to calibrate the distribution of our statistic: the asymptotic and the permutation method. In order to test which of the two approaches performs better, a simulation experiment has been performed considering different sample sizes, number of simulations, and permutations. The experiments indicate that the permutation method performs better than the asymptotic one given that power estimates approach closer to the significant value under the null hypothesis for low sample sizes. However, both approaches yield good estimates when considering large sample sizes (**Table 1**). This is in agreement with theoretical expectations given that for large samples, their statistical distribution should be equivalent, as the permutation distribution should converge to the tabulated distribution [37].

The results indicate that (i) the permutation method tends to fit better to the significance level than the asymptotic approach when the null hypothesis is true (specially for low sample sizes), and (ii) computational requirements using permutation can be an issue when considering a large number of iterations (large sample sizes); in such situations, the asymptotic calibration method might be more convenient (**Table 1**).

As done in Samuels et al., we would assume that haplogroup mtDNA frequencies in controls are known (note that there exist hundred of human population studies carried out to a local, regional or continental scale where these frequencies are available, at least for the most common haplogroups). We then simulated increases in the frequency of a risky haplogroup in cases, with the differences distributed proportionally between the remaining haplogroups (therefore, assuming there is no *a priori* assumption of an association with any of the remaining haplogroups considered) [26].

In agreement with Samuels et al.[26], we observed that power strongly depends on sample sizes, haplogroup population frequencies, and the deviation from the null hypothesis when using equal numbers of cases and controls (see solid lines in **Figure 1**). We next evaluate the situation where the number of cases differs from the number of controls. As shown in **Figure 1**, statistical power strongly depends on the case:control ratio when the other parameters are fixed, but this dependence is not as simple. As expected, power can increase very substantially as more controls exist relative to the number of cases. For instance, the statistical power to detect an association of haplogroup J increases from 60% to 80% when doubling the number of controls respect to cases in the example provided in **Figure 1**.

Samuels et al. [1] introduced the *N_scaled_* parameter for the estimation of the power. This parameter considers the difference between haplogroup frequency for equal numbers of controls and cases:



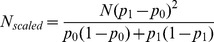
(1),

being *p_0_* the frequency of the risky haplogroup in controls, *p_1_* the frequency of the risky haplogroup in cases, and *N* the number of cases and controls (the total sample size is 2*N*).

We further consider the more general situation where the number of cases (N_ca_) can differ from the number of controls (N_co_),



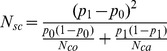
(2).

*N_scaled_* and *N_sc_* measures the squared standardized difference between frequencies in cases and in controls for the risky haplogroup. For 2×2 tables and a sample size large enough, the *N_scaled_* parameter follows a chi-square distribution with one degree of freedom due to the asymptotic normality of the standardized difference between frequencies [38].

**Figure 2:**
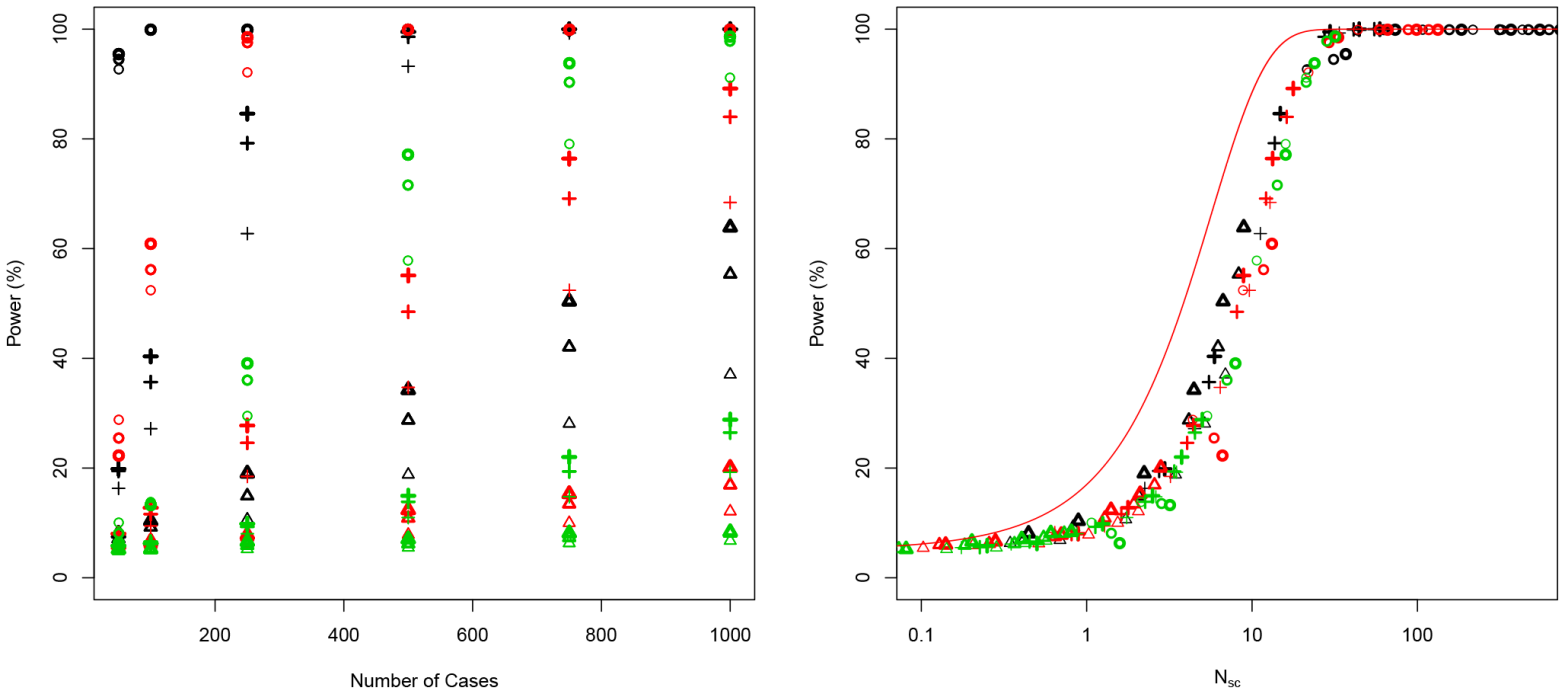
On the left side are the power values (α = 0.05) as a function of the number of cases for different haplogroups (the circles refer to haplogroup H, triangles to haplogroup J and crosses to haplogroup I). The intensity of the symbols indicate different odds ratio control-case: the thinner symbols indicate odds 1∶1, the medium symbols odds 2∶1, while the bolded symbols odds 3∶1. Colors indicate different deviations from the null hypothesis; black: the frequency of the risky allele is 100% higher in cases than in controls, red: 50%; and green 25%. The graph indicates that there is not a relationship between the number of cases and the statistical power value. On the right side are the power values as a function of the statistic *N_sc_*; in red is the theoretical curve for the statistical power for 2×2 tables when the number of controls is equal to the number of cases.

**Figure 3:**
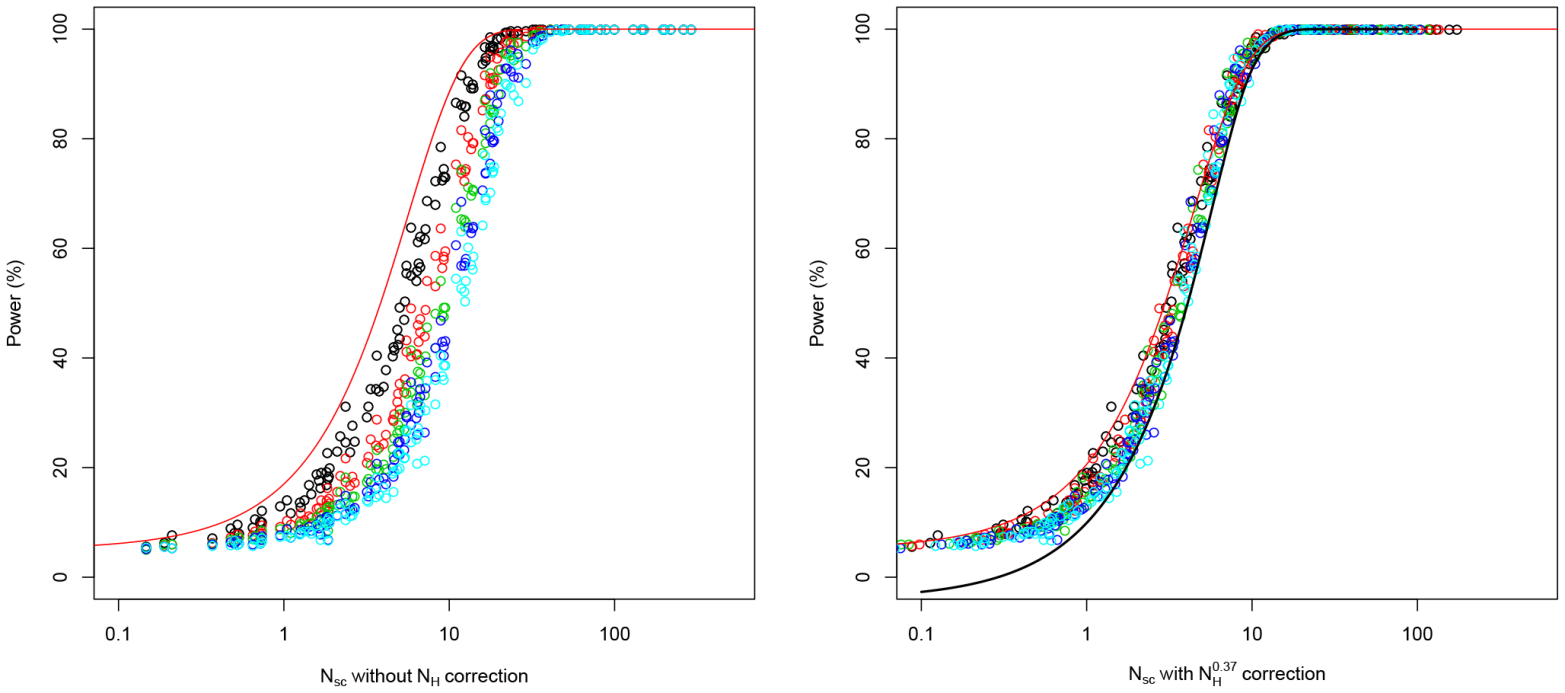
The left side shows power values (α = 0.05) as a function of *N_sc_* without correction of number of haplogroups (*N_H_*) for different number of haplogroups and when the number of cases equals the number of controls. The right side shows power corrected according to *N_H_*, and the nonparametric estimated regression curve. Colored circles denote different number of haplogroups; black: 4 haplogroups; red: 8 haplogroups; green: 12 haplogroups; dark blue: 16; and light blue: 20. Haplogroup frequencies were built using a vector of probabilities where the risk allele takes values 0.30, 0.15 or 0.05 (other values led to the same results; data not shown). The risky haplogroup take relative frequency differences in cases with respect to control of 100%, 50% and 25%. The number of cases takes values of 100, 250, 500, 750 and 1000, and control-case odds of 1, 2 and 3. We noted that other values do not change the distribution. The red line indicates the theoretical curve for 2×2 tables and equal numbers of cases and controls, while the black line is the non-parametric estimator of regression between *N_sc_* parameter and the statistical power.



As shown in **Figure 2**, there is a clear relationship between the parameter *N_sc_* and the power values. These values follow the theoretical curve obtained for 2×2 tables and equal numbers of cases and controls using the *arcsin* transformation [39]:
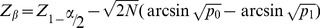
(3)Where *p_0_* in the frequency in controls, *p_1_*is the frequency in controls, *N* is the sample size for each arm and 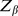 and 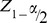 are normal quantile for 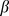 and 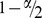.Simulations also indicate that the statistical power decreases as more haplotypes are tested (**Figure 3**). Samuels et al. introduced a parameter, *N_H_* (number of different haplogroups), that raised to the power of 0.37 allows to fit the statistical power to a single theoretical curve. According to Samuels et al. *N_scaled_* can be redefined as a function of the number of haplogroups analyzed:

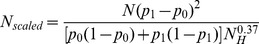
(4).

**Figure 4:**
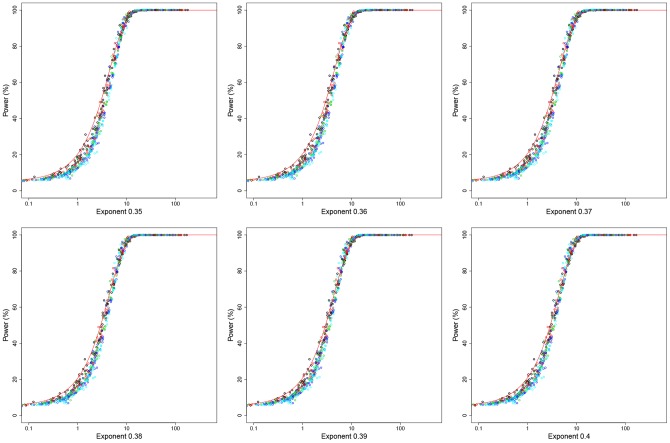
The simulations show that the parameter *N_H_* raised to the power of 0.37 is the one that empirically allows a better fit of the data to the theoretical curve of statistical power even in scenarios where the number of cases differs from the number of controls. See legend of **Figure 3** for more information on the simulation.

Note that the value 0.37 seems to have been obtained empirically by Samuels et al, (no specific formulae or indications were given in this regard). In the analysis shown in **Figure 4** we aimed to reproduce their findings. The simulations corroborate the fact that 0.37 is the value that allows to better fit the data to the theoretical curve for values of statistical power above 50%. Below 50% an exponent of 0.5 would perform better although it can assume that values of statistical power below 50% might be not relevant in association studies. Therefore, we observed that *N_H_* raised to the power of 0.37 allows fitting the statistical power to those scenarios where the number of cases differs from the number of controls (**Figure 3**).

Finally, *N_sc_* allows to relate all the parameters involved in the computation of the statistical power:
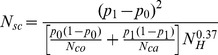
(5)


Samuels et al. (see A1 in their Appendix A) propose to use *N_scaled_* to estimate directly the statistical power and to determine the minimum number of controls and disease cases (*N_Cmin_*) required for a specific level of power (their formula (2)). However, this formula applies when number of cases equals the number of controls. The simulation method aims to overcome the limitation of this formula allowing for different sample sizes in cases and controls.

We then adjusted the parameter *N_Cmin_* for the more general scenario involving unequal numbers of cases and controls. Two options are possible: (i) estimation of the sample size given a control-case ratio; or (ii) estimation of the minimum number of controls (cases) when the number of cases (controls) is fixed.

In the first situation, if *N* denotes the number of cases, 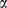 the control-case ratio, and 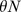 the number of controls, the minimum number of cases needed to reach a power 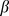 with a significance level 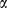 (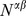) can be estimated from (5) as follows:
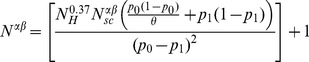
(6)where 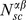 denotes the 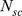 value providing a desired power 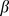 and a significance level 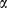, while [·] denotes the integer part function. The number of controls can be estimated as 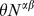. Note that a value of 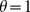
would reproduce the particular scenario considered by Samuels et al. [26].

In the second situation, the minimum number of controls (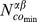) or the minimum number of cases (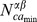) given a significance level 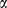 and a power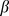, can also be estimated when the number of cases or the number of controls is fixed, respectively: 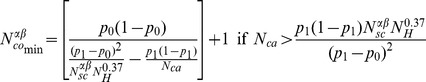
(7)




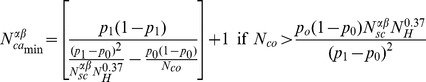
(8)where 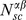 is the value providing the power desired 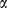 for a significance level 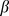, and [·] denotes the integer part function. This allows estimating the number of controls (number of cases) needed to reach a required power given a number of cases (number of controls). Note that statistical power is limited by the restrictions in equations (7) and (8); this is the reason of why power becomes stationary when the number of controls increases in regards to the number of cases (**Figure 1**).

It is also worthwhile to estimate the minimum deviation of the null hypothesis that can be detected for a power value 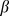 and a significance level 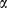 (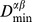), assuming we know haplogroup frequencies in controls and considering a given number of cases and controls. We can express 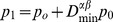 being 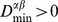 the deviation of null hypothesis. Note that it must verify 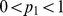. If we calculate from (1), it results (for a risky haplogroup):



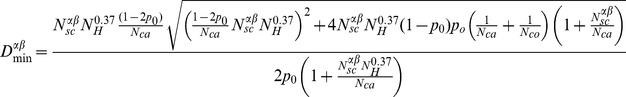
(9)


This difference can be expressed in terms of odds ratio. Thus

If 
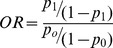
 then,
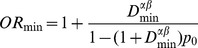
(10)Where *OR_min_* denotes the minimum OR that can be detected for a power value 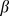 and a significance level 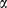.

The applications above require knowledge of the 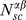
value given a significance level 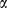 and a power 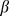. This parameter can be obtained by way of simulations and nonparametric regression (**Figure 3** [right] shows the scenario where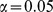). Note that nonparametric regression seems to perform well for power values above 60%. The package *sm* [40] implements local linear estimation, window-selector cross validation and Gaussian kernel, that allows to obtain 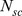 values for different significance levels α and power values. These values can be used in equations (6)–(10) in order to estimate the desired parameters (as done in **Table 2**).

**Table 2:** 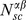
 estimates for a significance level α and a power value β using non-parametric regression.

β\α	10%	5%	1%	0.5%	0.1%
95%	11.61	13.79	18.68	19.84	21.33
90%	6.87	8.12	10.91	12.03	15.37
85%	5.97	7.07	9.47	10.64	13.77
80%	5.29	6.35	8.50	9.55	12.42
75%	4.59	5.80	8.05	8.86	11.42
70%	4.17	5.30	7.44	8.18	10.83
65%	3.72	4.68	6.82	7.84	10.15
60%	3.26	4.28	6.36	7.28	9.49
55%	2.96	3.88	5.96	6.75	9.79
50%	2.62	3.50	5.61	6.71	8.27

These values were averaged over ten simulations each. Note that these values differ slightly from those obtained by Samuels et al. (see their Table 3) [26] due to the simulation procedure implemented in both studies and because we consider unequal number of cases and controls.

The same simulation methods proposed to compute *a priori* statistical power can be applied for the estimation of the *a posteriori* power. Note however, that we treat *a posteriori* power in a different context as interpreted by others [41], [42]. Our procedure involves generating new data (tables) using the sample proportions and sample sizes obtained from a particular study. Therefore, the null hypothesis is tested by simulating new contingency tables. The procedures are analogs to the ones used to compute the *a priori* power.

Computation of the statistical power is essential to anticipate if the positive findings obtained in case-control disease studies are reliable. The present study has been motivated by the fact that the only available procedures to date to compute statistical power in mtDNA association studies [26] only allows to deal with scenarios involving 2×2 tables (or 2×3 tables), or if 2×*k* tables only study designs considering equal numbers of cases and controls (which does not represent the most common scenario in association studies). In the present study, we also provide with a web interface that implements the procedures developed in the present study (mitPower).

## Conclusions

During the last decade, a large number of mtDNA case-control studies have been published in the literature, most of them pointing to a number of haplogroups presumably associated with a complex disease. The validity of many of these conclusions might be questionable, given that most of them are underpowered. Most of these studies did not estimate the *a priori* statistical power because statistical tools were not available at the time.

The procedures developed in the present study allow the computation of statistical power in common as well as complex case-control study designs involving 2×*k*. The results indicate that underpowered studies could reach reasonable power by increasing the number of controls and reducing the number of hypothesis testing (i.e. haplogroups). In order to reach a wide range of researchers, we provide a friendly web-based tool (mitPower) that implements all the statistical procedures developed in the present study; this software can be used in both retrospective and prospective case-control disease studies. Note that the term retrospective is considered here as done before: “*the prospective power that can be obtained ignoring the fact that data have been gathered and a hypothesis has been tested. In essence, it computes the prospective power of the test as if: (a) the study and analyses had not yet been conducted and (b) the sample effect size is the hypothesized population effect size*” [43]. Further developments of mitPower could involve the implementation of multiple test corrections for the computation of the statistical power (in the sense as it was suggested before in 2×2 tables [44]) and in two-stage case-control designs [45]. Also challenging would be to explore the phylogenetic relationship existing between different haplogroups (the phylogenetic dependence) or mtSNPs and how this dependence could influence the estimation of power. Finally, other statistical/computational approaches could find their place in 2×*k* tables, such as the use of Markov Chain Monte Carlo methods (MCMC), already explored for 2×2×2 tables [46].

## Supporting Information

Figure S1 **Representation of power values for three haplogroups (H, J. and I) as a function of the number of cases and using the Fisher's exact test instead of the Chi-square test (** **Figure 1** **)(significance level of α = 0.05).** Colors indicate different deviations from the null hypothesis; thus, black represents a frequency in cases 100% higher than in controls, red represents an increment of 50%, and green an increment of 25% (with the difference distributed proportionally between the remaining non-risky haplogroups). The different lines indicate different case-control odds. The continuous line denotes an odd control-case of 1∶1, the dotted line of 2∶1, and the pointed line of 3∶1. Frequencies in controls for each haplogroup are indicated above each plot.
(TIF)

Table S1 **Comparison of different software packages for the estimation of statistical power and sample size estimation.**
(DOCX)
