# Supplementary figures and images for: A Generalized Model to Estimate the Statistical Power in Mitochondrial Disease Studies Involving 2×k Tables
Source: PLoS One. 2013 Sep 27;8(9):e73567. doi: 10.1371/journal.pone.0073567 (PMC3785462; doi:10.1371/journal.pone.0073567)

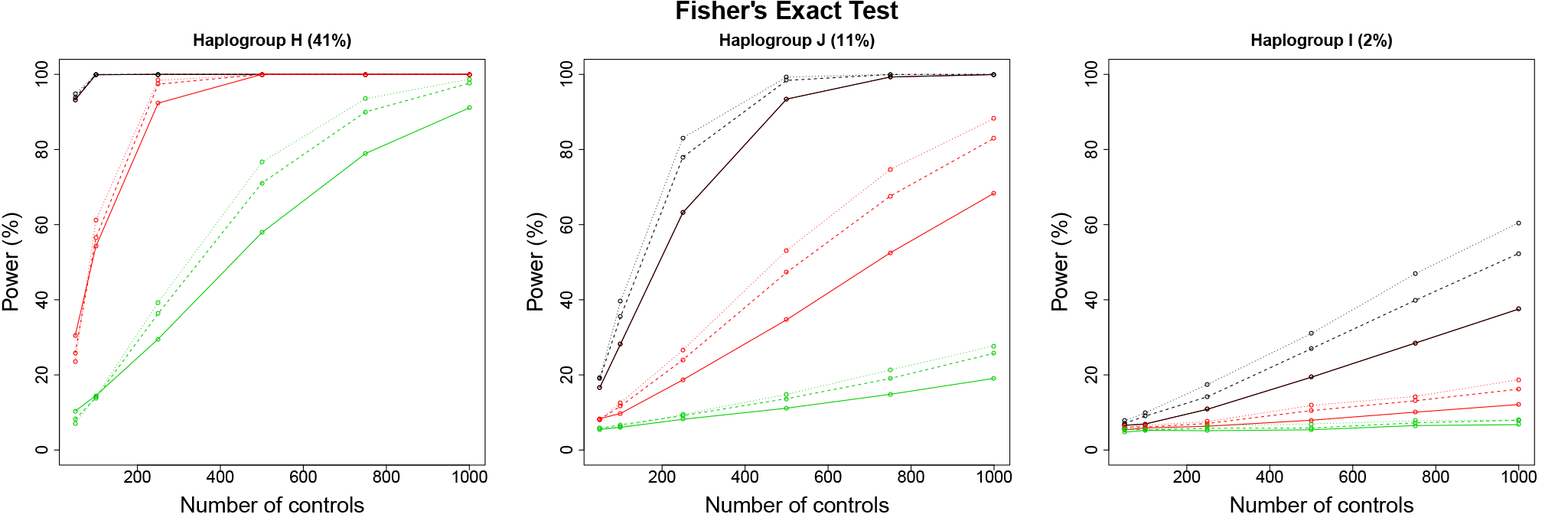

Supplement: Figure S1 — Representation of power values for three haplogroups (H, J. and I) as a function of the number of cases and using the Fisher's exact test instead of the Chi-square test ( Figure 1 )(significance level of α = 0.05). Colors indicate different deviations from the null hypothesis; thus, black represents a frequency in cases 100% higher than in controls, red represents an increment of 50%, and green an increment of 25% (with the difference distributed proportionally between the remaining non-risky haplogroups). The different lines indicate different case-control odds. The continuous line denotes an odd control-case of 1∶1, the dotted line of 2∶1, and the pointed line of 3∶1. Frequencies in controls for each haplogroup are indicated above each plot. (TIF) [file pone.0073567.s001.tif]
