# Supplementary material for: A Generalized Model to Estimate the Statistical Power in Mitochondrial Disease Studies Involving 2×k Tables
Source: PLoS One. 2013 Sep 27;8(9):e73567. doi: 10.1371/journal.pone.0073567 (PMC3785462; doi:10.1371/journal.pone.0073567)
Supplement: Table S1 — Comparison of different software packages for the estimation of statistical power and sample size estimation. (DOCX) [file pone.0073567.s002.docx]

**Table S1.** Comparison of different software packages for the estimation of statistical power and sample size estimation.

|  | | **1** | **2** | **3** | **4** | **5** | **6** | **7** | **8** | **9** | **10** | **11** | **12** | **13** | **14** |
| --- | --- | --- | --- | --- | --- | --- | --- | --- | --- | --- | --- | --- | --- | --- | --- |
| Features | Power Estimation | ✓ | ✓ | ✓ | 🗶 | ✓ | 🗶 | ✓ | ✓ | ✓ | ✓ | ✓ | ✓ | ✓ | ✓ |
|  | Sample size Calculator | ✓ | ✓ | ✓ | ✓ | ✓ | ✓ | ✓ | ✓ | ✓ | ✓ | ✓ | ✓ | 🗶 | ✓ |
| Contingency tables | 2 × 2 | ✓ | ✓ | ✓ | ✓ | ✓ | ✓ | ✓ | ✓ | ✓ | ✓ | ✓ | ✓ | ✓ | ✓ |
|  | 2 × *k* | ✓ | ✓ | 🗶 | 🗶 | 🗶 | 🗶 | 🗶 | 🗶 | 🗶 | 🗶 | 🗶 | 🗶 | ✓ | ✓ |
| Sample size to estimate power | Controls equals cases | ✓ | ✓ | ✓ | - | ✓ | - | ✓ | ✓ | ✓ | ✓ | ✓ | ✓ | ✓ | ✓ |
|  | Controls do not equal cases | 🗶 | 🗶 | ✓ | - | 🗶 | - | 🗶 | ✓ | ✓ | 🗶 | ✓ | ✓ | ✓ | ✓ |
| Sample size given a desired power | Controls equals cases | ✓ | ✓ | ✓ | ✓ | ✓ | ✓ | ✓ | ✓ | ✓ | ✓ | ✓ | ✓ | 🗶 | ✓ |
|  | Controls-case odds | 🗶 | 🗶 | ✓ | ✓ | 🗶 | ✓ | 🗶 | ✓ | 🗶 | 🗶 | ✓ | ✓ | 🗶 | ✓ |
|  | Any sample sizes in cases and controls | 🗶 | 🗶 | 🗶 | 🗶 | 🗶 | 🗶 | 🗶 | ✓ | 🗶 | 🗶 | 🗶 | ✓ | 🗶 | ✓ |
| Availability | Freeware tool | ✓ | 🗶 | ✓ | ✓ | ✓ | ✓ | ✓ | ✓ | ✓ | 🗶 | ✓ | ✓ | ✓ | ✓ |

**1**: G*Power 3 [[1](#_ENREF_1)]; **2**: Pass 12 [[2](#_ENREF_2)]; **3**: Quanto [[3](#_ENREF_3)]; **4**: Sample Size Calculator ; **5**: power.prop.test (R library; http://stat.ethz.ch/R-manual/R-patched/library/stats/html/power.prop.test.html); **6**: Proportion Difference Power/Sample size Calculation (http://statpages.org/proppowr.html); **7**: Power and Sample Size Programs (http://www.stat.ubc.ca/~rollin/stats/ssize/); **8**: Stplan (https://biostatistics.mdanderson.org/SoftwareDownload/SingleSoftware.aspx?Software_Id=41); **9**: StatsToDo (Sample Size for Two Proportions Program; https://www.statstodo.com/StatsToDoAbout.php); **10**: SigmaXL (http://www.sigmaxl.com); **11**: Sampsize (http://sampsize.sourceforge.net); **12**: pwr_2p.test (R library: http://rss.acs.unt.edu/Rdoc/library/pwr/html/pwr.2p2n.test.html); **13**: osDesign (R library; [[4](#_ENREF_4)]); **14**: mitPower (present study)

**References**

1. Faul F, Erdfelder E, Lang AG, Buchner A (2007) G*Power 3: a flexible statistical power analysis program for the social, behavioral, and biomedical sciences. Behav Res Methods 39: 175-191.

2. Hintze J (2013) PASS 12. NCSS, LLC. Kaysville: Utah, USA.

3. Gauderman WJ (2002) Sample size requirements for matched case-control studies of gene-environment interaction. Stat Med 21: 35-50.

4. Haneuse S, Saegusa T, Lumley T (2011) osDesign: An R Package for the Analysis, Evaluation, and Design of Two-Phase and Case-Control Studies. J Stat Softw 43.
